# An automated derivative-based method for detection of motor evoked potential onset latencies in multi-muscle transcranial magnetic stimulation studies

**DOI:** 10.1038/s41598-026-61560-0

**Published:** 2026-07-23

**Authors:** Rowan Boyles, Mikal Vicente, Sofia Zibordi, Jason Mallabone, Paul H. Strutton

**Affiliations:** 1 The Nick Davey Laboratory, Department of Surgery and Cancer, Imperial College London, London, UK; 2 Centre for Vestibular Neurology, Department of Brain Sciences, Imperial College London, London, UK; 3 Imperial College Healthcare NHS Trust, London, UK

**Keywords:** Transcranial magnetic stimulation, Motor cortex mapping, Motor evoked potentials, Upper limb, MEP latency, Automated detection, Neurology, Neuroscience

## Abstract

Motor evoked potential (MEP) onset latency is a useful neurophysiological measure, but manual measurement is time-consuming in transcranial magnetic stimulation (TMS) studies with large numbers of trials. This is particularly relevant in cortical mapping studies recording from multiple muscles simultaneously, where automated methods could support more scalable analyses. Existing onset-detection methods have shown promise in more restricted datasets, but their performance in heterogeneous multi-muscle mapping data remains uncertain. In this pilot validation study, three healthy adults underwent TMS cortical mapping with simultaneous EMG recording from eight upper-limb muscles during resting and active conditions, yielding 3,840 EMG epochs. Three independent raters classified MEP presence and marked onset latency for all trials. Inter-rater agreement was assessed using Fleiss’ κ for detection and ICC(2,1) for latency. Human majority vote for MEP presence and mean latency ratings were used as the reference standard to benchmark a novel derivative-ratio algorithm against an existing method. Human raters showed moderate to strong agreement for MEP detection (Fleiss’ κ = 0.69) and high reliability for latency ratings (ICC(2,1) = 0.95), with a pooled mean absolute pairwise difference of 0.88 ms. The derivative-ratio algorithm showed strong detection performance and human-like latency estimates, outperforming a previously published algorithm. These pilot validation data suggest that the derivative-ratio method provides promising automatic MEP onset latency detection in complex multi-muscle cortical mapping data and may provide a basis for scalable latency analysis. By enabling reproducible extraction of conduction-related MEP features, this approach may support future biomarker studies in neurological disorders characterised by altered corticospinal excitability or corticospinal conduction, pending further validation in larger and clinically diverse datasets.

**Supplementary Information:**

The online version contains supplementary material available at 10.1038/s41598-026-61560-0.

## Introduction

Transcranial magnetic stimulation (TMS) is an established neurophysiological technique with clinical and research applications. TMS uses rapidly changing magnetic fields to non-invasively induce electrical activity in central and peripheral nerves^1^ and can be used for both research and treatment purposes^2^. In studies of motor control, TMS is typically delivered over primary motor cortex to elicit motor evoked potentials (MEP) in different muscles, depending on the precise site of stimulation, measurable via electromyography (EMG). This enables characterisation of motor cortex excitability, corticospinal drive and conduction velocity, amongst other things^3^.

MEP onset latency is the time between stimulation and the start of the evoked potential and indicates the speed of conduction along the motor tracts. This includes central and peripheral components; activation of motor cortex interneurons followed by transmission along corticospinal tract and lower motor neurons to muscle fibres. MEP latencies provide information about the conduction of action potentials and the neuronal pathways involved in motor control^3^. This can be important in the characterisation and diagnosis of different neurological disorders. For example, slower central conduction velocity is characteristic of multiple sclerosis^4^ and damage to the corticospinal tract in spinal cord injury or stroke leads to longer MEP latencies^5–7^.

Nevertheless, measurement of onset latencies is time-consuming, particularly for MEP studies with large numbers of trials. As such, there is interest in automatic detection methods, and several algorithms have been developed to detect onsets of neurophysiological parameters including MEP latencies and silent periods^8–12^. These typically identify the MEP onset based on the EMG signal rising above a particular threshold level, such as a proportion of the pre-stimulus EMG. For example, Daskalakis et al. defined MEP onset as the last crossing of the mean pre-stimulus EMG level by the squared EMG signal, before the point of maximum deflection^12^. Huang et al. used the time point at which the EMG signal exceeded the pre-stimulus EMG by more than 5 standard deviations^10^. Soda et al. describe the squared hard threshold estimator (SHTE), which determines onset by finding the point where the squared EMG signal crosses the threshold level of 10% of the maximal amplitude, and then subtracting a constant value from this^13^. Such threshold-based methods facilitate latency detection but are less accurate than manual ratings^14^.

A novel method was more recently developed and tested by Bigoni et al. on MEPs measured from the resting first dorsal interosseous (FDI) muscle in healthy controls and patients with stroke^14^. This algorithm used the derivative of the EMG signal (the change in slope of the EMG waveform) to detect onsets and reported more accurate measurements than previous methods, in line with ‘gold-standard’ human-rated latencies. In brief, this method restricts analysis to a fixed post-stimulus window, identifies the principal MEP deflection after taking the absolute value of the signal, and then calculates the first derivative of the waveform up to this deflection. MEP onset is defined as the first sample in the longest consecutive sequence of samples with a positive derivative, thereby identifying the start of the sustained rising phase of the response, rather than relying on a hyperparameter, such as a fixed amplitude threshold. This is an important step in automation of TMS data analysis but requires further validation in studies involving measurements from multiple muscles or under conditions of muscle activity where ongoing background activity may make detection of slope changes more difficult. Motor control of the upper limb depends on coordinated activity across multiple proximal and distal muscles rather than the isolated behaviour of a single muscle^15–18^. Recording MEPs from several muscles simultaneously may therefore provide a more functionally relevant picture of corticospinal output and cortical organisation, particularly in studies seeking to characterise patterns of muscle co-activation relevant to everyday movement.

One useful application of automatic latency detection is in TMS cortical mapping studies. These build a picture of cortical excitability by delivering TMS repeatedly over an area around the motor hotspot for a given muscle. This enables more detailed characterisation of the organisation of motor cortex through individual muscle metrics such as area and centre of gravity^19,20^. Mapping studies involve large numbers of stimuli, in excess of 80 per map^21^ and may measure from multiple muscles simultaneously^22,23^. Previous studies have investigated the topography of MEP latencies, finding for example that they increase with increasing distance from the hotspot^19,24^. Whilst an automated MEP onset latency detection algorithm would therefore be particularly useful for cortical mapping studies, it must be robust to handle the variations in MEP size, shape and latency that are observed when generating cortical maps, especially when recording from multiple muscles simultaneously^25^.

The present pilot validation study arose from efforts to develop a detection method that is robust to these variations and to test it in a diverse cortical mapping dataset, comparing it to manual reference ratings and an existing automated method^14^. The Bigoni algorithm was chosen as a comparator due to its established high accuracy relative to other automated methods^14,26^. Although more recent data suggest that a neural-network (NN) based detector, DELMEP, offers high accuracy^26^, this was not evaluated here because its reported training and validation were limited to intrinsic hand muscles. Deployment in a heterogeneous multi-muscle mapping dataset would therefore ideally require application-specific pretraining, particularly given expected differences in latency distributions across muscles. In contrast, the algorithm described here is directly deployable without study-specific training data, which may be advantageous for diverse cortical mapping datasets. Furthermore, since Bigoni et al.’s algorithm has shown broadly comparable performance to DELMEP in previous benchmarking^26^, it represents a suitable comparator.

## Methods

### Participants

For the recording of MEPs, three healthy right-hand-dominant adults (2 female, 1 male; mean ± SD age = 26 ± 3 years) were recruited from the investigators’ local network as part of a larger study developing rapid mapping protocols for clinical application. All procedures conformed to the Declaration of Helsinki and were approved by the Imperial College London Research Ethics Committee (ref: 6386803). Written informed consent was obtained from each participant. For the measurement of MEP latencies, three researchers with different levels of experience carried out manual measurements of the latencies of MEPs recorded from cortical mapping EMG data (see below for details).

### Electromyography (EMG)

Disposable Ag/AgCl surface-electrode pairs (inter-electrode distance ≈ 20 mm) were placed on eight muscles of the dominant upper limb: first dorsal interosseous (FDI), abductor pollicis brevis (APB), abductor digiti minimi (ADM), extensor digitorum communis (EDC), flexor digitorum superficialis (FDS), biceps brachii (BB), triceps brachii (TB) and anterior deltoid (AD). A single reference electrode was positioned over the contralateral ulnar styloid. Signals were amplified (×1000) and band-pass filtered (10–1000 Hz; Digitimer D360, Welwyn Garden City, UK), then digitised at 2 kHz via a Power 1401 A/D converter (Cambridge Electronic Design [CED], Cambridge, UK) and streamed to a PC running CED Signal for real-time monitoring and storage.

### Transcranial magnetic stimulation (TMS) protocol

Stimulation was delivered with a Magstim 200^2^ connected to a 70 mm figure-of-eight coil (The Magstim Company, Whitland, UK). Coil location and orientation were tracked with a Brainsight neuronavigation system (BrainBox Ltd, Cardiff, UK) using the MNI template warped to individual cranial landmarks (nasion and pre-auricular points). The FDI hotspot was identified over contralateral M1 (left side for all participants) as the scalp position eliciting the largest peak-to-peak amplitude of the MEP from FDI. Resting motor threshold (RMT) was defined as the lowest intensity evoking ≥ 3 out of 6 MEPs ≥ 0.05 mV. A 6 × 6 cm grid centred on the hotspot (edge parallel to the longitudinal fissure) was mapped with a pseudo-random “random-walk”^21^ sequence of 80 stimuli (0.5 Hz) at each of two conditions:

- *Rest*: Intensity = 1.8 × RMT.
- *Active*: Intensity = 1.2 × RMT with added isometric contraction. Participants flexed their shoulder to 90°, fully extended elbow and splayed fingers.

This protocol was originally developed to optimise rapid cortical mapping from multiple muscles simultaneously and to examine the effect on cortical maps of adding activity to a resting mapping protocol. As such, several methodological decisions were intended to increase efficiency and ensure comparability across conditions; namely, using FDI hotspot to centre the grid, thresholding once for FDI only and using RMT, rather than separately determining active motor threshold (AMT) for the active condition.

### Dataset used for algorithm evaluation

Two maps (one active, one resting) were created for each participant (80 stimuli × 8 muscles = 1280 EMG epochs). Across three participants this yielded 3840 EMG trials in total. Individual trials comprised a 250 ms pre-trigger baseline and 500 ms post-stimulus window. DC offset was removed in post-processing as required.

### Derivative-ratio algorithm

#### Summary of algorithm process

The algorithm first detects the peak of each MEP, then searches back through the trace to identify the earliest time point where the signal starts to climb steeply. At every step it compares how fast the signal is increasing before that point with how fast it is rising after it; when the rise-after clearly outweighs the rise-before, that point becomes a candidate onset latency. A final sanity check keeps only candidates that are physiologically plausible (e.g. occur < 35 ms after the stimulus and are followed by a consistently steep slope). Anything that fails these checks is marked “undetected”. Values for algorithm variables were derived from initial testing on separate EMG data which did not form part of the algorithm evaluation presented here.

The derivative-ratio detector was implemented as custom Python code (version 3.13.5), and all automated detections reported in this study were generated using this implementation. Standard scientific Python libraries were used for numerical processing and data handling. The script was run offline on single-trial EMG epochs and returned either an onset latency in milliseconds or an “undetected” classification for each trial.

#### Algorithm process in detail

##### Amplitude gate

An epoch enters MEP onset latency detection if the peak to peak amplitude in the 5–45 ms window post-stimulus exceeds 1.1 × the peak-to-peak amplitude of the baseline (–100 to − 1 ms), and if the peak occurs sufficiently close to the peak measured on the average of all epochs in a given condition/muscle (within ± 15 ms of average peak).

##### Derivative-ratio back-tracking scan

* k* : sample index under evaluation.

* B* : block length (5 samples − 2.5 ms given a sampling rate of 2 kHz).

$\:\varDelta\:ptp$ : peak-to-trough distance within the MEP window (no. of EMG samples).

- Anchor – Locate the first extremum (peak or trough) 5–45 ms post TMS.

$$\text{Anchor index}=\:k0$$.

b) Search limits – Scan backwards from * k0* to * kmin*.

$$\:\mathrm{kmin}=\text{max of either}\:k0\:-\:(1.75\:\times\:\:\varDelta\:ptp)\quad \text{or 5ms post-TMS}$$

This maintains the search window within a plausible time scale of the MEP peak and prevents interference from any post-stimulus artefact. Ratio of $\:1.75\:\times\:\:\varDelta\:ptp$ determined from initial testing.

c) Derivative-ratio – For each *k* in that range compute ratio R: 
  $$\:R\left[k\right]=\:\frac{\mu\:fwd}{\mu\:bwd+\epsilon\:}$$
   

where: $\:\mu\:fwd\:=\:$ mean absolute first derivative in the B samples after $\:\mathrm{k}$. $\:\mu\:bwd\:=\:$ mean absolute first derivative in the B samples before $\:\mathrm{k}$. $\:\epsilon\:\:=\:{10}^{-6}$ , added to the denominator to avoid division by zero.

d) Candidate block – Let * Rmax* be the largest ratio; collect the contiguous block of indices with $\:R\left[k\right]\ge\:0.85\:\times\:\hspace{0.17em}Rmax$. The earliest of these becomes the primary candidate. This means that for any cluster of large ratios (e.g. if the onset is slightly more gradual) the earliest point will be selected.

##### True MEP filter

To ensure that the candidate onset is associated with something resembling a genuine MEP, candidate latency * k* is accepted when all criteria hold:

1. Latency cap – * k* < 35 ms post-stimulus. This upper bound was selected to retain the expected range of upper-limb MEP onset latencies while excluding late post-stimulus fluctuations unlikely to reflect the initial corticospinal response. Because proximal upper-limb muscles generally have shorter MEP latencies than distal hand muscles, this cap was conservative for proximal responses and primarily served to prevent late noise or secondary waveform components from being misclassified as MEP onset.
2. Initial slope – Derivatives of ≥ 3 of the first 4 samples after k exceed baseline mean derivative for current epoch (ensures that captures genuine, consistent slope).
3. Overall slope – For the window after * k* (width $\:\varDelta\:ptp\:\times\:2$), check that mean derivative is greater than baseline mean derivative + 1.5 SD (ensures that overall slope of section after onset is much greater than baseline, indicating a likely MEP).

If no candidate onset passes, the trace is labelled as undetected.

#### Benchmarking against manual ratings

Three independent raters, all authors (one doctoral researcher, one MRes student and one research physiotherapist) reviewed every EMG epoch in a custom graphical interface (either CED Signal or an equivalent Python viewer). For each channel they:

1. Flagged the trace as MEP present or absent.
2. When present, positioned a cursor at the onset as identified visually.

Raters were permitted to zoom and pan traces to aid visual placement of the onset cursor. However, no additional filtering, smoothing or signal transformation was applied during manual review. Raters were blind to one another and to algorithm output. Muscle order and contraction condition were presented exactly as acquired, mimicking real-world measurements in clinical or research settings.

#### Inter-rater reliability

##### MEP detection (binary–present/absent)

Fleiss’ κ was calculated across the three raters to quantify chance-corrected agreement.

##### Latency (continuous)

Absolute-agreement ICC(2,1) assessed concordance of single latency ratings. Per-muscle ICCs were computed within each muscle/condition, and human pairwise mean absolute differences, bias and limits of agreement (LoA) were pooled across rater pairs.

These values informed the appropriateness of averaging the human ratings to serve as a reference standard for comparison to the algorithm. To combine ratings, for each epoch the majority vote for MEP detection was determined (2 or 3 raters agree) and mean latency calculated if MEP was determined to be present.

### Algorithm performance

Using the human consensus reference:

- MEP Detection: Sensitivity, specificity, precision, F1-score, and Cohen’s κ for (i) the derivative-ratio detector, and (ii) the Bigoni method^14^.
- Latency: Mean absolute error (MAE), root-mean-square error (RMSE) and Bland–Altman bias/limits for both algorithms versus the human mean.

All statistics were performed in Python 3.13.5 using statsmodels, pingouin, and scikit-learn; figures were produced in Matplotlib.

### Sensitivity analyses

To provide a fair comparison between the two algorithms, two sensitivity analyses were run. Firstly, although the EMG in this experiment was recorded at 2 kHz, the Bigoni algorithm was validated on 5 kHz EMG. Since it relies on finding a sequence of positive derivatives, a lower sampling rate reduces the number of consecutive data points and may disadvantage this method. As such the 2 kHz EMG data were resampled to 5 kHz before latency detection using polyphase finite impulse response filtering within the scipy library in Python.

Secondly, because the dataset included proximal and distal muscles, latency measurements varied from between ~6ms to ~ 27ms. As such, the detection window for algorithms was set to start at 5ms. However, the Bigoni algorithm was originally designed to detect MEPs within a 10-50ms window. To determine the impact of onset window on algorithm accuracy, the Bigoni algorithm was re-run with a 10ms onset. Proximal muscle MEPs with onsets < 10ms would not be detected.

To examine the effect of empirically derived heuristics (such as search window and block length for ratio calculation) on algorithm performance, further sensitivity analyses were run with different values of these variables. The results of these are provided in the supplementary materials.

### Computational runtime

**Fig. 1:**
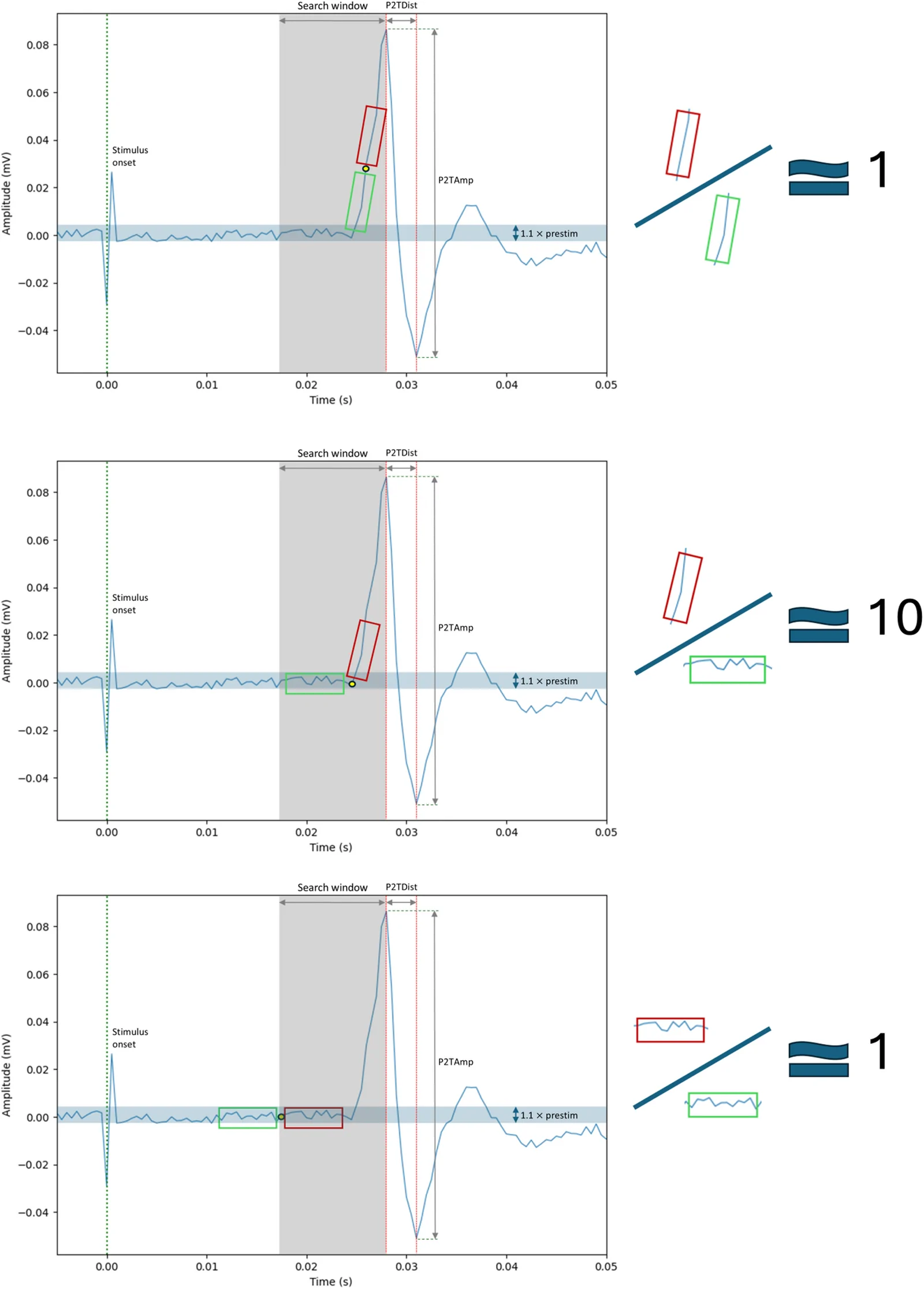
Schematic representation of the derivative-ratio latency detection procedure. Values and window positions are illustrative and are not drawn to scale. Traces were first screened for a detectable MEP; epochs were retained if the post-stimulus peak-to-trough amplitude (P2TAmp) was ≥ 1.1 times the pre-stimulus P2TAmp (blue shading). The algorithm then identified the first MEP extremum (peak or trough) and searched backwards through the search window, shaded in grey (1.75 x P2TDist). At each sampled time point, the mean first derivative was calculated over a 2.5 ms window before the point and after the point. The derivative ratio was defined as the post-point derivative divided by the pre-point derivative. Points lying on a relatively constant slope therefore produced ratios close to 1, whereas points at the transition from baseline to the rising MEP produced a larger ratio. The point producing the largest ratio defines the candidate onset region.

Computational runtime for both algorithms was measured on a Windows system with an AMD Ryzen 9 7940HS processor (8 cores/16 threads) and 32 GB RAM. The code was run in Spyder using Python 3.13.5, with no GPU acceleration. Elapsed wall-clock time was recorded using Python’s time.perf_counter() from loading each epoched EMG file to completion of CSV export. These data are provided in the supplementary materials (Fig. 1).

## Results

### Inter-rater reliability (reference standard)

**Fig. 2:**
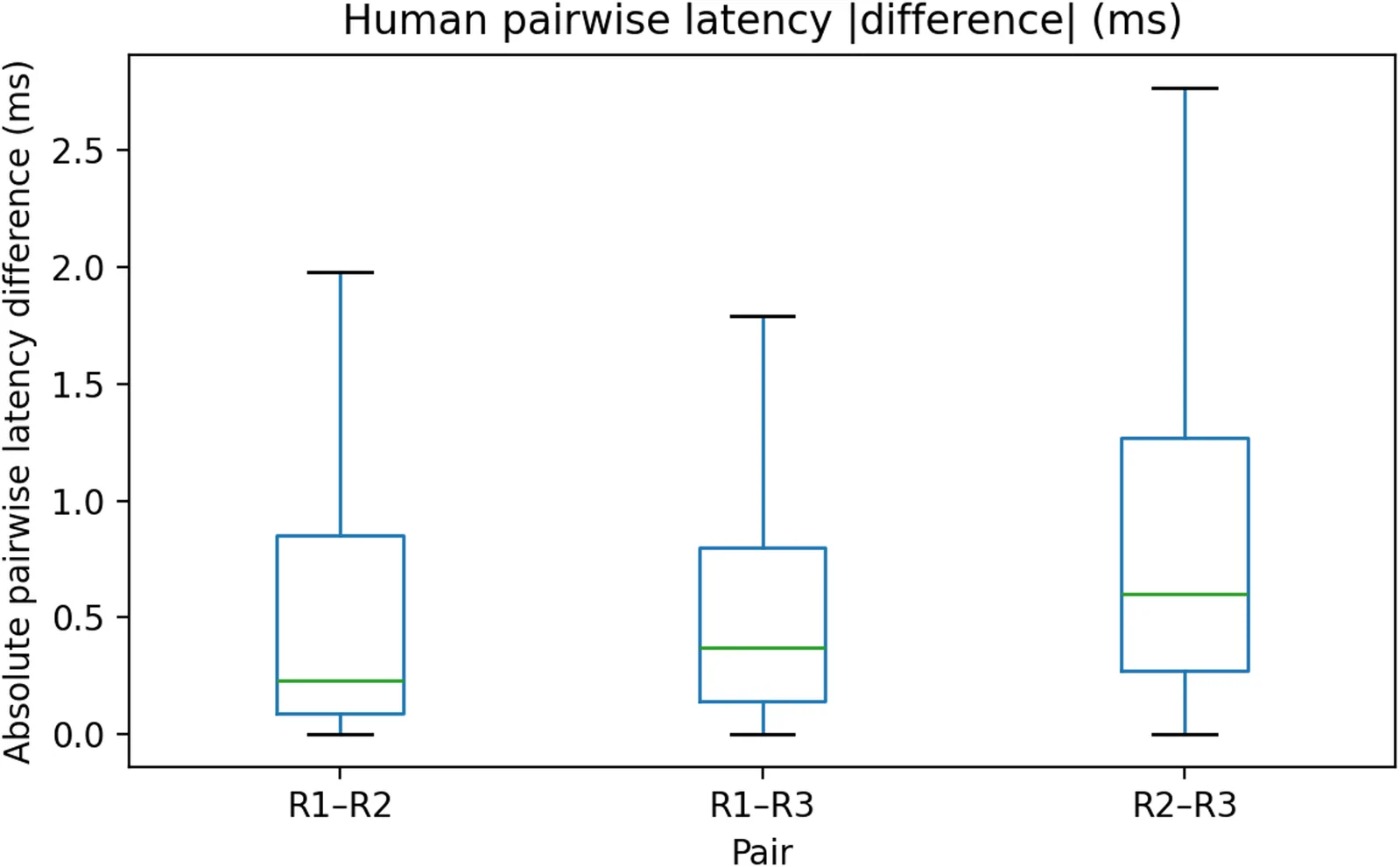
Box plot of pairwise latency differences for each rater pair. Boxes indicate 25th (Q1) to 75th (Q3) percentile of differences. Green line = median pairwise difference. Whiskers indicate 1.5 x IQR below Q1 and above Q3.

For MEP detection, Fleiss’ κ was 0.69 across 3840 epochs indicating moderate to substantial agreement. For latency, relative agreement was high with ICC(2,1) = 0.95 across 2773 targets rated by all three raters. Across all rater pairs (*N* = 8597 overlapping measurements), the pooled mean absolute difference was 0.88 ms, with a pooled bias of − 0.08ms, and 95% limits of agreement (LoA) from − 3.16ms to + 3.00ms. Per-pair biases were − 0.39ms (R1–R2), − 0.15ms (R1–R3), and + 0.31 ms (R2–R3). By muscle, distal hand muscles (FDI, APB, ADM) had the highest ICCs, while some proximal groups (e.g., FDS, TB) had lower ICCs, especially during active recordings. Human raters therefore showed substantial detection and latency agreement. As such, the three raters’ scores were averaged to create the reference onset. Majority vote was used for MEP presence, arithmetic mean was used for latency. Consensus presence occurred in 3051 epochs (2773 with 3/3 present, 278 with 2/3 present); all other epochs were treated as consensus absent (Fig. 2).

**Table 1:** Human rater ICC and errors per muscle. The top half of the table shows results from resting muscles. The bottom half shows results from active muscles. N indicates the number of epoch pairs used for the calculation of these. *Pairwise mean absolute differences.

	FDI	APB	ADM	EDC	FDS	TB	BB	AD
Resting MEPs								
N	698	714	700	704	696	378	493	392
ICC(2,1)	0.86	0.91	0.89	0.65	0.45	0.30	0.62	0.50
Abs. diff. (ms)*	0.64	0.55	0.52	0.50	0.58	1.22	0.78	1.80
Bias(ms)	− 0.02	0.13	0.05	0.24	0.09	− 0.56	− 0.28	− 1.32
LoA(ms)	[− 1.88, 1.84]	[− 1.34, 1.60]	[− 1.64, 1.73]	[− 1.12, 1.61]	[− 1.70, 1.89]	[− 4.75, 3.63]	[− 3.32, 2.76]	[− 6.30, 3.67]
Active MEPs								
	FDI	APB	ADM	EDC	FDS	TB	BB	AD
N	493	474	494	584	494	398	390	495
ICC(2,1)	0.80	0.73	0.76	0.28	0.47	0.36	0.49	0.47
Abs. diff. (ms)*	0.80	0.75	1.03	0.91	0.95	1.44	1.09	1.51
Bias(ms)	− 0.13	0.00	0.00	0.21	-0.11	− 0.08	− 0.10	− 0.19
LoA(ms)	[− 2.68, 2.42]	[− 2.52, 2.52]	[− 3.68, 3.67]	[− 2.79, 3.21]	[− 3.37, 3.15]	[− 4.47, 4.32]	[− 3.88, 3.68]	[− 4.95, 4.58]

Although the pooled across-muscle inter-rater reliability was high, within-muscle ICCs were lower (e.g. FDS/TB/BB notably modest; active < rest; Table 1). This pattern is expected because ICC is a ratio of variance components and is attenuated by range restriction: within a given muscle the true latency range is narrow, so the same small rater differences constitute a larger share of total variance, lowering ICC. In other words, modest ICCs can coexist with small absolute disagreements. Because ICC depends on the spread of true scores, it is not directly comparable across muscles/conditions with very different latency ranges; hence mean absolute difference and LoA are reported here as complementary, scale-interpretable measures.

### Algorithm vs. human consensus

**Table 2:** Summary of algorithm latency detection accuracy vs. human consensus measurements. Original 2 kHz dataset.

	Derivative- ratio: overall	Derivative- ratio: rest	Derivative- ratio: active	Bigoni: overall	Bigoni: rest	Bigoni: active
True positive	2808	1557	1251	1789	926	863
True negative	605	186	419	677	249	428
False positive	184	93	91	112	30	82
False negative	243	84	159	1262	715	547
Sensitivity	0.92	0.95	0.89	0.59	0.56	0.61
Specificity	0.77	0.67	0.82	0.86	0.89	0.84
Precision	0.94	0.94	0.93	0.94	0.97	0.91
F1	0.93	0.95	0.91	0.72	0.71	0.73
MAE(ms)	1.06	0.99	1.14	2.74	2.36	3.15
RMSE(ms)	1.59	1.46	1.74	4.63	3.93	5.28
Bias(ms)	− 0.31	− 0.45	− 0.13	− 1.91	− 1.65	− 2.19
SD(ms)	1.56	1.38	1.74	4.22	3.57	4.80
LoA(ms)	[− 3.36, 2.75]	[− 3.17, 2.60]	[− 3.53, 3.27]	[− 10.18, 6.36]	[− 8.65, 5.36]	[− 11.60, 7.23]

The derivative-ratio method demonstrated good detection (Cohen’s κ = 0.67; F1 = 0.93) with strong sensitivity and moderate specificity, and produced human-like latency measurements (MAE = 1.06ms, bias = − 0.31ms; Table 2). The Bigoni method was much more conservative demonstrating high precision but lower sensitivity, missing around 41% of genuine MEPs (Cohen’s κ = 0.29; F1 = 0.72). It also showed a systematic early bias (− 1.91ms) and the latency MAE (2.74ms) was outside the human pairwise band; limits of agreement were much wider than the ratio method (Table 2). Performance was marginally better in rest than active for both humans and algorithms. Human and algorithm agreement was better in hand muscles than proximal muscles (Tables 3 and 4).

**Fig. 3:**
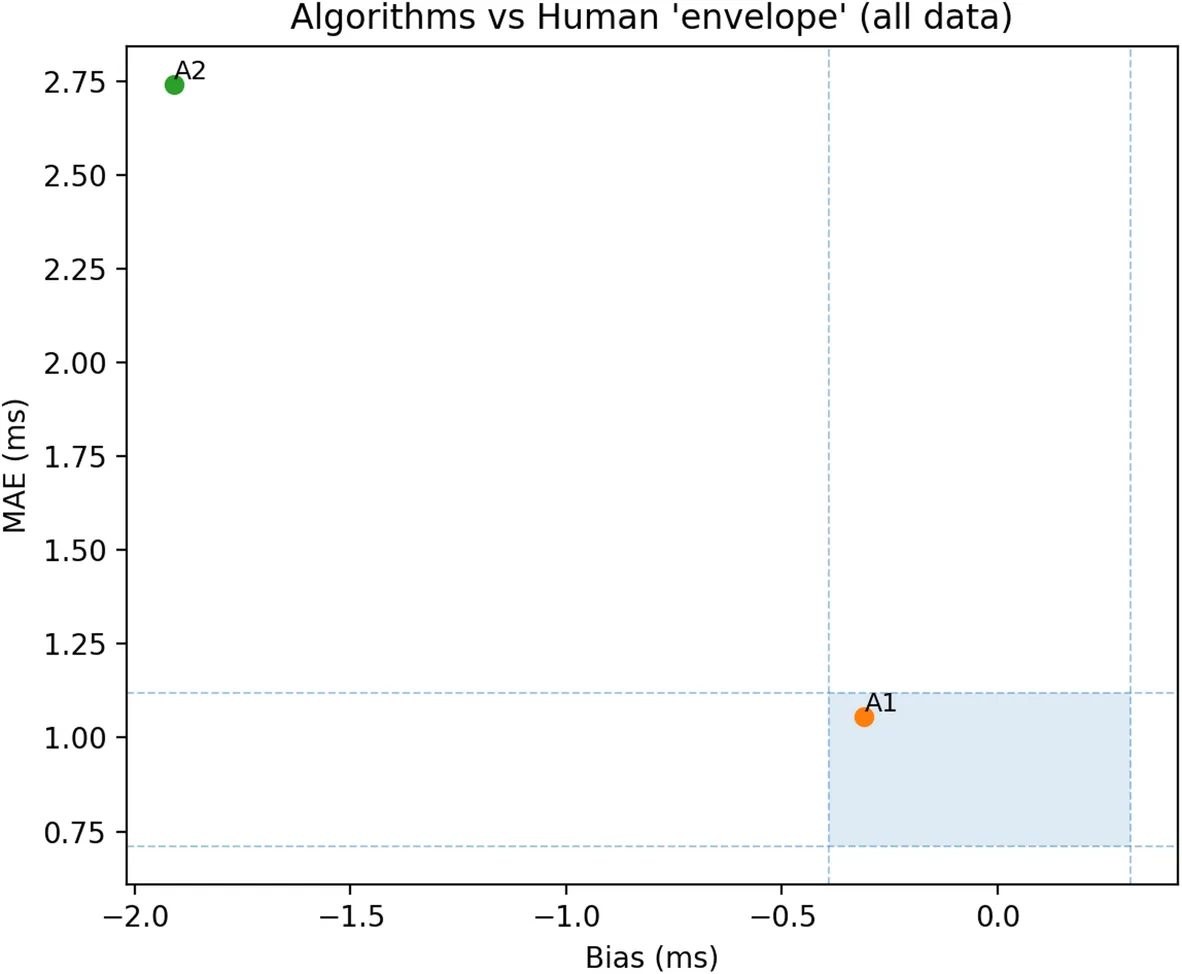
Algorithms vs. human envelope (bias–MAE). Bias (ms; algorithm − human consensus) on the x-axis and MAE (ms) on the y-axis. The shaded rectangle shows the exact human–human envelope: its vertical edges are the minimum and maximum pairwise human bias, and its horizontal edges are the minimum and maximum pairwise human MAE. Dashed lines coincide with these edges. Points inside the rectangle have both bias and error within human–human variability (positive bias = later latencies). A1 = derivative-ratio, A2 = Bigoni.

**Table 3:** Summary of algorithm latency detection accuracy vs. human consensus measurements, by muscle – resting map.

Muscle	FDI	APB	ADM	EDC	FDS	TB	BB	AD
Algorithm	Derivative-ratio							
N	229	232	226	227	226	122	172	123
ICC(2,1)	0.77	0.79	0.81	0.65	0.29	0.46	0.54	0.69
MAE(ms)	0.95	0.67	0.98	0.53	0.84	1.86	1.30	1.48
Bias(ms)	− 0.45	− 0.26	− 0.61	− 0.45	− 0.59	− 1.36	− 0.23	0.29
LoA(ms)	[− 2.90, 2.00]	[− 2.48, 1.97]	[− 2.83, 1.60]	[− 1.83, 0.93]	[− 2.54, 1.37]	[− 5.18, 2.46]	[− 3.96, 3.50]	[− 3.38, 3.96]
Algorithm	Bigoni							
N	137	160	114	156	134	78	68	79
ICC(2,1)	0.22	0.26	0.02	0.23	− 0.02	0.29	0.45	0.45
MAE(ms)	4.20	2.10	3.13	1.12	1.70	2.14	2.26	2.43
Bias(ms)	− 4.13	− 1.72	− 2.52	− 1.11	− 0.88	− 0.65	− 0.51	− 0.23
LoA(ms)	[− 13.82, 5.56]	[− 8.27, 4.82]	[− 11.36, 6.33]	[− 3.74, 1.52]	[− 5.61, 3.85]	[− 6.00, 4.69]	[− 6.49, 5.46]	[− 6.45, 5.99]

**Table 4:** Summary of algorithm latency detection accuracy vs. human consensus measurements, by muscle – active map.

Muscle	FDI	APB	ADM	EDC	FDS	TB	BB	AD
Algorithm	Derivative-ratio							
N	168	166	156	197	162	135	125	142
ICC(2,1)	0.71	0.63	0.70	0.29	0.45	0.36	0.55	0.51
MAE(ms)	0.92	0.95	1.41	0.93	1.20	1.38	1.08	1.37
Bias(ms)	− 0.62	− 0.18	− 0.18	− 0.40	− 0.28	0.38	− 0.16	0.66
LoA(ms)	[− 3.22, 1.97]	[− 3.27, 2.90]	[− 4.27, 3.91]	[− 3.42, 2.61]	[− 3.31, 2.76]	[− 3.52, 4.29]	[− 3.17, 2.85]	[− 3.14, 4.46]
Algorithm	Bigoni							
N	135	126	106	127	80	116	67	106
ICC(2,1)	0.12	− 0.05	0.04	0.03	0.18	0.13	0.21	0.03
MAE(ms)	3.87	4.28	6.00	1.73	1.88	2.11	1.77	2.72
Bias(ms)	− 3.78	− 4.14	− 5.16	− 1.56	− 0.55	0.25	− 1.26	− 0.15
LoA(ms)	[− 12.42, 4.86]	[− 14.54, 6.26]	[− 17.77, 7.45]	[− 5.69, 2.57]	[− 6.84, 5.74]	[− 7.44, 7.95]	[− 5.77, 3.25]	[− 10.11, 9.82]

### Sensitivity analyses

As can be seen in Table 5, upsampling to 5 kHz substantially improved Bigoni performance. Shifting the window start from 5 to 10 ms reduced latency error and bias but slightly reduced sensitivity, consistent with the exclusion of very early proximal responses. The derivative-ratio algorithm remained more accurate overall. Further details of sensitivity analyses including full per-muscle breakdowns are available in the supplementary materials.

**Table 5:** Summary of algorithm latency detection accuracy vs. human average measurements, sensitivity analysis Derivative ratio 2 kHz results are repeated here for comparison.

	Derivative-ratio 2 kHz, 5ms onset	Bigoni 5 kHz, 5ms onset	Bigoni 5 kHz, 10ms onset
True Positive	2808	2619	2591
True Negative	605	579	577
False Positive	184	210	212
False Negative	243	432	460
Sensitivity	0.92	0.86	0.85
Specificity	0.77	0.73	0.73
Precision	0.94	0.93	0.92
F1	0.93	0.89	0.89
MAE(ms)	1.06	2.21	1.65
RMSE(ms)	1.59	4.01	2.84
Bias(ms)	− 0.31	− 1.11	− 0.19
SD(ms)	1.56	3.85	2.84
LoA(ms)	[− 3.36, 2.75]	[− 8.65, 6.44]	[− 5.77, 5.38]

## Discussion

Whilst automation of amplitude measurement is relatively straightforward and accurate in modern signal processing software, latency detection is harder and until recently, relied on manual methods. Manual rating requires ~ 10 s per trial, which for the present dataset entailed ~ 10 h of repetitive measurement for each rater. As such, developments in signal processing offer useful ways to facilitate feasible, large scale, clinically-relevant latency analyses. The Bigoni method uses the derivative of the EMG signal to identify the start of the sustained rising phase of the MEP^14^ and offers accuracy similar to human raters – a significant improvement over methods that rely on hyperparameters such as thresholds of the prestimulus EMG^9,12^, or maximum amplitude^13^. However, it has only previously been evaluated in resting intrinsic hand muscles with an approximate MEP latency range of 20-30ms^14,26,27^, potentially limiting applicability to data from different muscles or under conditions of activity.

The present study examined automatic MEP latency measurement in a heterogeneous TMS cortical mapping dataset, comprising both resting and active EMG from multiple upper limb muscles with diverse latency profiles. MEP latencies for these muscles have previously been reported to range from < 10ms to around 35ms^27–31^. Detection was consistent between human raters and latency measurements were reliable with only small differences between rater pairs. When compared to the human average, the derivative-ratio algorithm provided robust, human-like MEP onset latency detection. It performed less well in active conditions and in proximal muscles, but this was consistent with human raters where a decrease in reliability and consistency of latency measurements was also observed. The derivative-ratio algorithm performed better than the Bigoni algorithm. One weakness of the Bigoni method was its highly conservative MEP detection, leading to high specificity but very low sensitivity. It also had greater errors in latency estimates, a bias towards earlier onsets and much wider limits of agreement, putting its results outside of the observed range of human raters (Fig. 3).

Sensitivity analyses intended to provide a fairer comparison demonstrated that the Bigoni algorithm performed better with data upsampled to 5 kHz. Increasing the detection window from 5ms to 10ms improved the MAE and RMSE. Sensitivity was improved as were accuracy and bias. Since the Bigoni method relies on finding a minimum number of consecutive samples with a positive derivative, at a lower sampling rate the signal has fewer data points over the same time period. This affects the derivative calculation and could potentially make it less likely to find a long, uninterrupted chain of rising samples. Despite this, the limits of agreement remained wide and the derivative ratio method was more accurate overall.

The reasons for the poorer performance of the Bigoni algorithm are not clear. It appears that relying on a chain of positive derivatives sometimes fails to detect the transition from background EMG to MEP. Although this metric does typically correspond to MEP onset, in polyphasic MEPs, the longest chain may capture the rise to the maximum amplitude *after* an initial smaller peak. This would lead to a later onset being marked. In situations with larger background fluctuations, such as during muscle activity, spurious early peaks may result in long positive derivative chains. This may explain the particular difficulty of the Bigoni algorithm with active EMG data in this study (see supplementary data for further details). Accurate latency detection under active conditions is important in motor control studies since proximal muscles have been shown to respond more readily in active conditions than with increased stimulator intensity^25^. Since the derivative ratio is designed to detect the point of maximum deflection preceding the maximum, it is less likely to capture spurious peaks and represents an important refinement of derivative-based detection methods.

The present findings should also be interpreted in relation to other recent approaches to automated MEP analysis. Traditional threshold-based methods, including SHTE^13^, have the advantage of being simple and transparent, but depend on empirically chosen thresholds or offsets and may be sensitive to baseline activity, amplitude and waveform morphology. DELMEP represents a novel and promising direction, using deep learning to estimate MEP latency and showing excellent accuracy in large, annotated datasets^26^. However, this also distinguishes it from the present approach, which was designed as a deterministic, explainable detector that can be applied without pre-training. MEPFeatX further illustrates the growing need for accessible tools for automated MEP feature extraction, including latency, duration and polyphasia^32^. However, because MEPFeatX is primarily a feature-extraction package rather than a dedicated validation study of latency detection against human onset ratings, it was not included as a direct comparator here.

Beyond methodological performance, automated MEP onset latency detection may support emerging biomarker pipelines by providing a scalable conduction-related measure that complements amplitude-, threshold- and excitability-based TMS metrics. This may be relevant in disorders characterised by altered corticospinal excitability or conduction, including ALS, stroke, epilepsy syndromes and other motor-system diseases. Recent studies in ALS and cortical hyperexcitability disorders highlight the increasing use of neurophysiological measures for stratification, prognosis and treatment-response monitoring^33–35^. Within this context, automated latency extraction could enable reproducible integration of MEP latency with MEP amplitude, CMAP, clinical phenotype, functional decline and fluid biomarkers across multiple muscles, stimulation sites and repeated assessments.

### Limitations

A key limitation is the small validation sample, with data derived from only three healthy participants. Although this yielded a large number of EMG epochs for benchmarking, repeated epochs from a small participant sample cannot fully account for broader between-participant variation. The findings should therefore be interpreted as a preliminary validation of the detector, rather than definitive evidence of generalisability across healthy and clinical populations.

Nevertheless, the dataset was heterogeneous at the level most directly relevant to automated latency detection: the recorded EMG waveform. MEPs were recorded from eight upper-limb muscles, spanning distal and proximal representations, both at rest and during contraction, and across multiple stimulation sites rather than only at a single hotspot. This produced substantial variation in latency, amplitude, signal-to-noise ratio, background activity and waveform morphology, including polyphasic responses in some muscles. Example EMG waveforms are provided in the supplementary materials. The dataset therefore provides a valuable test of detector performance across diverse upper-limb MEP morphologies. Further validation remains necessary in larger samples and in clinical populations where delayed, dispersed or highly polyphasic responses may be more common.

A further limitation is that several algorithmic parameters were empirically selected. Although final evaluation was performed on data that did not form part of the initial parameter development, the block length, search-back window, amplitude gate and candidate threshold are heuristic choices. This creates a risk that some settings are partly tuned to the acquisition characteristics and waveform distributions encountered during development, including sampling rate, filtering, upper-limb muscle set, surface EMG configuration and healthy-participant physiology. The sensitivity analyses reported in the supplementary materials provide an initial assessment of parameter dependence. Future work should optimise and validate these parameters using larger annotated datasets with explicit training, validation and independent test partitions, ideally with cross-validation across participants, muscles, recording systems and clinical groups.

## Conclusion

Recording neurophysiological measurements from multiple muscles simultaneously can give insights into functional motor control, given that upper limb function involves complex muscle synergies. However, manual latency detection of MEPs is laborious and resource intensive. In this pilot validation study, the derivative-ratio method performed within the observed human-rater latency envelope and showed strong MEP detection performance with small bias, providing a practical route to scalable TMS mapping analyses. This could be valuable in the study of clinical populations where upper limb function is compromised and, more broadly, in biomarker pipelines requiring scalable, reproducible extraction of MEP-derived features across muscles, stimulation sites and repeated assessments.

## Supplementary Information

Below is the link to the electronic supplementary material.

Supplementary Material 1

## Data Availability

All Python code implementing the derivative-ratio detector, together with anonymised example EMG, is openly available at https://doi.org/10.5281/zenodo.16966802.
