# Supplementary material for: An automated derivative-based method for detection of motor evoked potential onset latencies in multi-muscle transcranial magnetic stimulation studies
Source: Sci Rep. 2026 Jul 23;16:26643. doi: 10.1038/s41598-026-61560-0 (PMC13507071; doi:10.1038/s41598-026-61560-0)
Supplement: Supplementary file 1 [file 41598_2026_61560_MOESM1_ESM.docx]

# Supplementary Materials

## A: Example MEP Traces

Supplementary Figure S1: Demonstrating intra-individual variation in MEPs. EMG traces representing the response in all muscles to a single TMS stimulus. X axis represents time in seconds, y axis voltage in millivolts. Left panel, participant 1, stimulation @ 1.8 x FDI RMT - resting. Right panel, participant 2, stimulation @ 1.2 x FDI RMT – active. All y axis scales are optimised for clarity. Note variations in latency, amplitude and morphology across muscles. FDI - first dorsal interosseous, APB - abductor pollicis brevis, ADM - abductor digiti minimi, EDC - extensor digitorum communis, FDS - flexor digitorum superficialis, BB - biceps brachii, TB - triceps brachii, AD - anterior deltoid

## B: Sensitivity Analyses

Supplementary Table S1: Sensitivity analysis results - derivative ratio parameter settings. Results for default settings in first row for comparison. Sens: Sensitivity; MAE: Mean Average Error, RMSE: Root Mean Squared Error. Ratio calc block = length of EMG block used to calculate derivative ratio. Nearby ratio threshold = proportion of max ratio used to include alternative timepoint as potential onset. PtT = peak to trough (of putative MEP). Effects of altering parameters were relatively small. The largest impact on performance was seen with a short ratio calculation block and long search windows. Further lengthening of the search window produced little additional change beyond the modest reduction in accuracy already observed at longer search settings.

| Derivative Ratio Parameter Setting | Sens | F1 | MAE | RMSE | Bias | Main change vs default |
| --- | --- | --- | --- | --- | --- | --- |
| Default Settings | 0.92 | 0.93 | 1.06 | 1.59 | -0.31 | — |
| Ratio calc block (short): 2 ms | 0.91 | 0.93 | 1.09 | 1.64 | -0.29 | Slightly less accurate |
| Ratio calc block (long): 3 ms | 0.92 | 0.93 | 1.04 | 1.55 | -0.34 | Minimal difference |
| Nearby ratio threshold (low): 0.75 | 0.92 | 0.93 | 1.06 | 1.59 | -0.31 | Minimal difference |
| Nearby ratio threshold (high): 0.95 | 0.92 | 0.93 | 1.05 | 1.58 | -0.31 | Minimal difference |
| Search window (short): 1.5 x PtT distance | 0.92 | 0.93 | 1.03 | 1.54 | -0.18 | Slightly less negative bias |
| Search window (long 1): 2.0 x PtT distance | 0.92 | 0.93 | 1.07 | 1.64 | -0.40 | Slightly more negative bias |
| Search window (long 2): 4.0 x PtT distance | 0.90 | 0.92 | 1.15 | 1.92 | -0.54 | Slightly less accurate, slightly more negative bias |
| Search window (long 3): 10.0 x PtT distance | 0.90 | 0.92 | 1.16 | 1.95 | -0.55 | Slightly less accurate, slightly more negative bias |

Supplementary Table S2: Per-muscle algorithm performance results for Bigoni 5 kHz, 5ms onset window.

| **Condition** | **Resting** | | | | | | | |
| --- | --- | --- | --- | --- | --- | --- | --- | --- |
| **Muscle** | **FDI** | **APB** | **ADM** | **EDC** | **FDS** | **TB** | **BB** | **AD** |
| **N** | 182 | 182 | 182 | 182 | 181 | 135 | 167 | 131 |
| **ICC(2,1)** | 0.26 | 0.21 | 0.00 | 0.44 | -0.00 | 0.35 | 0.63 | 0.63 |
| **MAE(ms)** | 3.47 | 1.96 | 2.56 | 0.73 | 1.39 | 1.97 | 1.18 | 1.82 |
| **Bias(ms)** | -3.02 | -1.29 | -1.18 | -0.66 | -0.40 | 0.20 | -0.21 | 0.38 |
| **LoA(ms)** | [-13.01, 6.96] | [-8.42, 5.84] | [-9.35, 7.00] | [-2.21, 0.90] | [-4.90, 4.10] | [-5.40, 5.80] | [-4.01, 3.58] | [-4.17, 4.92] |
| **Condition** | **Active** | | | | | | | |
| **Muscle** | **FDI** | **APB** | **ADM** | **EDC** | **FDS** | **TB** | **BB** | **AD** |
| **N** | 169 | 165 | 160 | 177 | 168 | 141 | 140 | 157 |
| **ICC(2,1)** | 0.18 | 0.01 | 0.04 | 0.15 | 0.17 | 0.17 | 0.23 | 0.04 |
| **MAE(ms)** | 2.98 | 3.47 | 4.55 | 1.34 | 1.97 | 1.92 | 1.61 | 2.46 |
| **Bias(ms)** | -2.63 | -3.00 | -3.16 | -1.12 | -0.60 | 0.66 | -0.47 | -0.19 |
| **LoA(ms)** | [-10.75, 5.48] | [-13.22, 7.23] | [-14.93, 8.61] | [-4.84, 2.60] | [-7.01, 5.80] | [-6.33, 7.66] | [-5.30, 4.37] | [-9.03, 8.64] |

Supplementary Table S3: Per-muscle algorithm performance results for Bigoni 5 kHz, 10ms onset window.

| **Condition** | **Resting** | | | | | | | |
| --- | --- | --- | --- | --- | --- | --- | --- | --- |
| **Muscle** | **FDI** | **APB** | **ADM** | **EDC** | **FDS** | **TB** | **BB** | **AD** |
| **N** | 180 | 182 | 182 | 182 | 181 | 135 | 168 | 122 |
| **ICC(2,1)** | 0.37 | 0.31 | 0.12 | 0.53 | 0.11 | 0.41 | 0.71 | 0.55 |
| **MAE(ms)** | 1.95 | 1.44 | 1.93 | 0.69 | 1.09 | 1.68 | 0.93 | 1.69 |
| **Bias(ms)** | -1.20 | -0.69 | -0.51 | -0.62 | -0.09 | 0.58 | 0.28 | 1.43 |
| **LoA(ms)** | [-7.10, 4.70] | [-5.31, 3.93] | [-6.27, 5.26] | [-1.82, 0.59] | [-3.06, 2.88] | [-4.27, 5.43] | [-2.52, 3.07] | [-1.64, 4.49] |
| **Condition** | **Active** | | | | | | | |
| **Muscle** | **FDI** | **APB** | **ADM** | **EDC** | **FDS** | **TB** | **BB** | **AD** |
| **N** | 170 | 166 | 160 | 177 | 168 | 142 | 134 | 142 |
| **ICC(2,1)** | 0.26 | 0.08 | 0.21 | 0.31 | 0.42 | 0.08 | 0.40 | 0.00 |
| **MAE(ms)** | 2.32 | 2.26 | 3.03 | 1.00 | 1.23 | 2.01 | 1.04 | 2.37 |
| **Bias(ms)** | -1.92 | -1.65 | -1.49 | -0.74 | 0.34 | 1.85 | 0.66 | 2.27 |
| **LoA(ms)** | [-7.90, 4.06] | [-8.70, 5.40] | [-10.00, 6.98] | [-3.17, 1.68] | [-3.40, 4.08] | [-4.22, 7.91] | [-2.30, 3.61] | [-5.29, 9.84] |

## C: Computational Runtime

Supplementary Table S4: Processing time required for both algorithms. Figures presented are mean averages of 10 runs for each algorithm on the evaluation dataset in this study, comprising 3840 epochs. “Total” indicates the total processing time for all 3840 epochs. “Frame” is the time taken to process a single frame of 8 channels. “Channel-frame” is the time taken to process a single channel in a single frame. Both algorithms have low processing time but the Bigoni algorithm was computationally more efficient and faster overall.

|  | Total (s) | Frame (ms) | Channel-frame (ms) |
| --- | --- | --- | --- |
| Derivative Ratio | 0.8891 | 1.842 | 0.231 |
| Bigoni | 0.1320 | 0.265 | 0.031 |
